# Ultra-high *Q*-factor and amplitude-tunable Fano resonance in vanadium dioxide–silicon hybrid metamaterials

**DOI:** 10.1039/d4ra01301h

**Published:** 2024-04-25

**Authors:** Kun Deng, Yachen Gao, Yang Gao, Tong Wu

**Affiliations:** a Key Laboratory of Medical Electronics and Digital Health of Zhejiang Province, Jiaxing University Jiaxing 314001 China wutongjx@zjxu.edu.cn; b Engineering Research Centre of Intelligent Human Health Situation Awareness of Zhejiang Province, Jiaxing University Jiaxing 314001 China; c Electronic Engineering College, Heilongjiang University Harbin 150080 China

## Abstract

As the resonance response in magnetic systems usually occurs at low frequencies, previously known as terahertz high *Q*-factor resonances, are mainly excited by electrical resonances. In this paper, we present a metamaterial based on vanadium dioxide–silicon arrays capable of achieving a *Q*-factor of up to 165 198; the ultra-high *Q*-factor Fano resonance excited by the proposed metamaterial is mainly affected by strong magnetic resonance. The analysis of diffractive coupling theory, electric field, magnetic field and current distribution shows that strong magnetic resonance is mainly realized by coupling localized plasmon resonance with the lattice resonance. Due to the conductivity-tunable nature of vanadium dioxide, the proposed metamaterial features an amplitude tunable function with a modulation depth of 98.8%. The spectral response of the analyte demonstrates the capability of the proposed metamaterial for application as a sensor with a maximum sensitivity of 69.52 GHz per RIU and a figure of merit of 15 456. The ultra-high *Q*-factor performance and amplitude tunability of the proposed structure can be applied to terahertz devices, such as ultrasensitive sensors, filters and optical switches.

## Abstract 2

We present a VO_2_-silicon metamaterial with *Q* factor up to 165 198 that can be used for terahertz sensing. By adjusting the conductivity of VO_2_, an optical switching function with a modulation depth of 98.9% can be realized.

## Graphical abstract

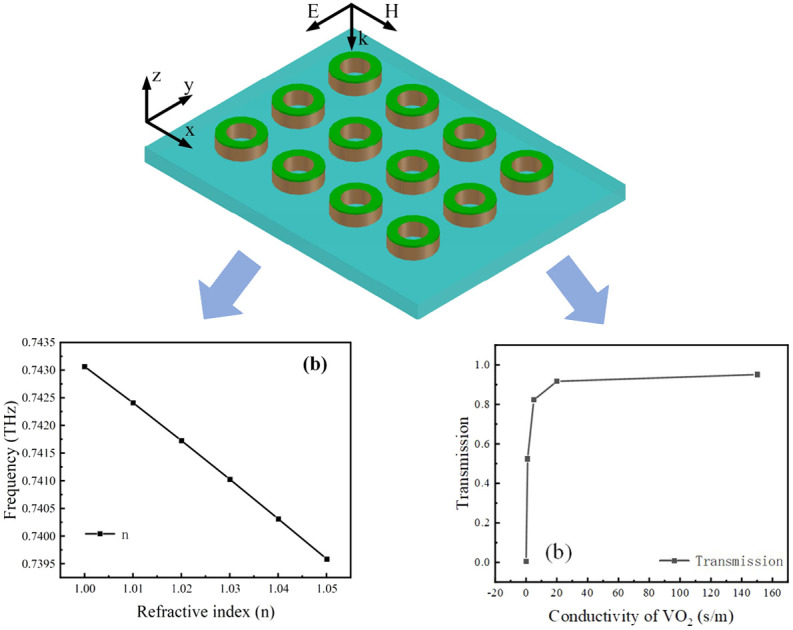

## 1. Introduction

Terahertz wave is a sub-millimeter wave in the frequency range of 0.1–10 THz. It has the characteristics of microwaves and far-infrared waves because it is in the range where both wave types overlap.^1,2^ In recent years, the development of terahertz technology has provided new means for the development of many disciplines, such as biology, chemistry, and physics.^3–5^ Terahertz sensing is an important research direction for the evaluation of terahertz sensing performance as well, where the quality factor (*Q*-factor) is an important parameter. The key to realizing a high *Q*-factor resonance is to reduce the dipole radiation loss.^6,7^ In plasmon structures, Fano resonance can reduce or even completely suppress radiation loss.^8^ It can produce an anomalous asymmetric and narrow bandwidth line shape through the interference of narrowband dark and broadband bright modes.^9^

The emergence of metamaterials provides an effective solution for the excitation of Fano resonance, which is characterized by the ability to artificially design the geometry of structural units.^10–12^ Enhanced manipulation of terahertz waves and the response of their fields to matter is possible by combining multiple materials and designing different cell structures.^13^ Metamaterial-excited Fano resonance has become an important research direction.^14–17^ With the development of tunable materials, such as graphene and VO_2_, multifunctional sensors based on metamaterials have progressed significantly.^18^ For example, in 2023, Xin *et al.* used a metal structure and GaAs to prepare a Fano-resonant metamaterial that could achieve a sensitivity of 513 GHz per RIU and a maximum modulation depth of 96.5% by an adjustment of the conductivity of GaAs.^19^ In 2023, Ma *et al.* designed an all-media metamaterial based on silicon and reported strong intrinsic chirality of the planar cross resonance and a high *Q*-factor for it.^20^ In the same year, Feng *et al.* proposed a metamaterial, whose unit structure consisted of asymmetric triple-parallel silicon rectangles and recorded a high *Q*-factor and high robustness for it.^21^ In 2023, Wang *et al.* proposed a two-band metamaterial absorber at terahertz frequency with a periodically patterned metallic structure on the top of the wafer, which exhibited perfect absorption properties and a high *Q*-factor in two bands, 0.89 THz and 1.36 THz.^22^ In 2023, He *et al.* proposed a four-band tunable narrow-band terahertz absorber that had a perfect multimodal absorption in the 1–9 THz range and could sense with high sensitivity.^23^ However, it is worth noting that as the resonant response in magnetic systems usually occurs at low frequencies, previously reported terahertz high *Q*-factor resonance was mainly excited by electrical resonances.^24^ There are still only a few metamaterials with tunable Fano resonances to achieve the ultra-high *Q*-factor, and most such metamaterials are sensitive to the polarization of the incident wave, limiting their application scenarios.

In this paper, we design an ultra-high *Q*-factor metamaterial that can be used for sensing and optical switching. The unit structure of this metamaterial consists of vanadium dioxide (VO_2_) and silicon rings that are periodically arranged and can be insensitive to incident polarization. In addition, the field distributions and currents of the proposed metamaterial at the resonance frequency indicate that the ring resonator is capable of exciting significant electric quadrupole resonance and magnetic dipole resonance. The diffraction coupling theory analysis concludes that significant second-order (1,1) surface lattice resonance (SLR) occurs at the Fano resonance frequency. The proposed metamaterial achieves extremely narrow Fano resonance peaks *via* coherent Fano interactions between the localized plasmon resonance (LPR) of individual ring resonators and SLR of the array. The far-field radiation losses are also suppressed due to the destructive interference of the metamaterial resonator with the scattered field of the substrate, resulting in ultra-high *Q*-factor Fano resonance. In terms of sensing performance, proposed metamaterials can achieve a sensitivity of 165 198 and a figure of merit (FoM) of 15 456. The Fano resonance peak amplitude is also modulated by changing the conductivity of VO_2_.

## 2. Structural design and methods

**Fig. 1:**
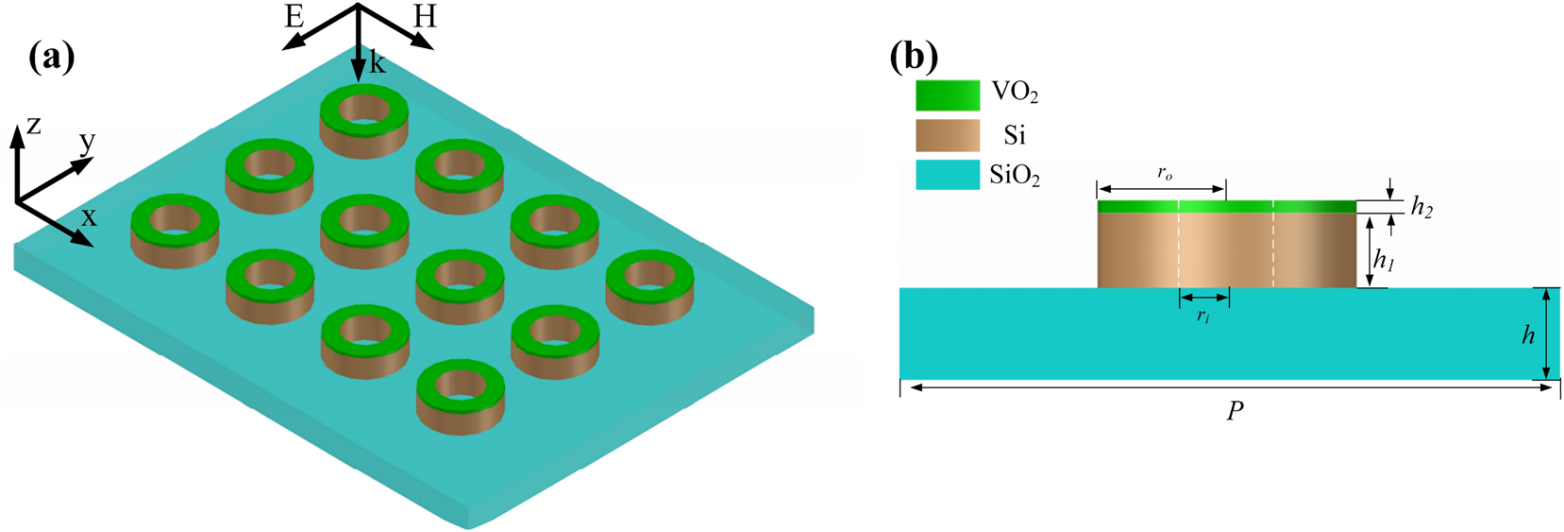
(a) Schematic and (b) front view of the structure of the VO_2_–silicon metamaterial.

Fig. 1 illustrates an overall schematic and the front view of the proposed metamaterial. The substrate consists of a 20 μm-thick silicon dioxide (SiO_2_) layer with a dielectric constant of 1.49.^25^ As a phase-change material, VO_2_ has a low carrier concentration, and it is an insulating material at a temperature of 28 °C. When the temperature is increased to 68 °C, VO_2_ reaches a conductivity of 200 000 S m^−1^ and achieves an insulating-to-metallic transition, which can occur at sub-picosecond rates.^26^ Compared to metal-based metasurfaces, dielectric-based metasurfaces have lower losses to the incident wave. Thus, the proposed resonator unit of the metasurface is realized by a combination of silicon and vanadium dioxide.^27^ The resonator unit on the substrate consists of silicon and VO_2_ rings, which are stacked and distributed in an array with a period of 400 μm. The inner and outer ring radius of the silicon and VO_2_ ring embraces are 50 μm and 75 μm and thicknesses are 35 μm and 0.2 μm, respectively. The permittivity of silicon is 3.45.^28^ The centrosymmetric feature of the proposed metamaterial unit enables it to possess the advantage of being insensitive to the electromagnetic field polarization of the incident wave.

The permittivity of VO_2_ can be obtained by *ε*(*ω*) = *ε*_∞_ − [*ω*_p_^2^(*σ*)/*ω*(*ω* + *iγ*)], where *ε*_∞_ = 12 is the high-frequency permittivity; *ω*_p_(*σ*) is the conductivity-dependent plasma frequency, and *γ*is the collision frequency. In addition, *ω*_p_(*σ*)and *σ* are proportional to the free carrier density. Therefore, the plasma frequency at *σ* frequency can be roughly expressed as *ω*_p_^2^(*σ*) = *σω*_p_^2^(*σ*_0_)/*σ*_0_, where *σ*_0_ = 3 × 10^3^ Ω^−1^ S^−1^, *ω*_p_(*σ*_0_) = 1.4 × 10^15^ rad s^−1^, and *γ* = 5.75 × 10^13^ rad s^−1^.^29,30^ Terahertz plane wave was positively incident along the *z*-axis to the proposed metamaterial. The *S* parameter can be calculated by the FDTD theory, and the transmission *T* can be obtained by *T* = |*S*_21_|^2^.^31^

## 3. Results and discussion

**Fig. 2:**
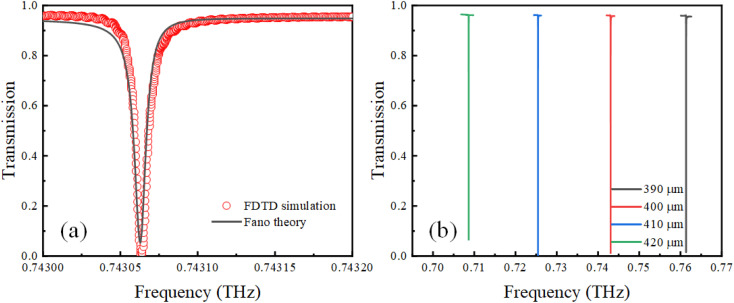
(a) Transmission spectra calculated by FDTD simulation and Fano theory, (b) transmission spectra with different metasurface periods.

The initial incident wave polarization was set to be in the *x* direction, and the initial conductivity of VO_2_ was 0. The transmission properties of the proposed metamaterial in the terahertz band were numerically calculated using the FDTD algorithm, and the results are shown in Fig. 2(a) as red circles. It can be observed that an asymmetric Fano curve with a narrow line width exists at 0.743063 THz. Furthermore, we analyzed Fano characteristics using eqn (1), where *a*_1_ + *ia*_2_ denotes the background transmission without the resonator and comprises real numbers; *γ* is the damping loss, and *ω*_0_ is the resonant frequency.1
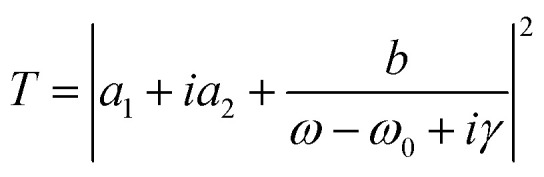


The fitting results are shown as the black line in Fig. 2. It can be seen that theoretical fitting results are in good agreement with numerical simulation results. Correspondingly, the *Q*-factor is approximately 165 198 from the formula, *Q* = Re(*ω*)/2Im(*ω*) = *ω*_0_/2*γ*.^32^

In this work, in-plane SLR could be generated in the metamaterial due to the periodic arrangement of the resonator units in the design. When the resonance frequency of a single resonator is close to the SLR frequency, a strong energy transfer can be achieved from the incident terahertz wave to the local plasma, resulting in a sharp plasma resonance.^33,34^

Based on the diffractive coupling theory,^35^ the resonant frequency of the SLR can be expressed as eqn (2).2
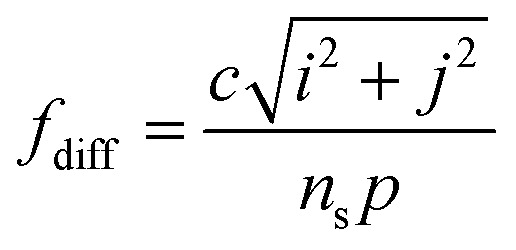
where *P* is the lattice constant of a periodic array, and (*i*, *j*) is a nonnegative integer defining the order of the SLR. In our work, the second-order (1,1) SLR has a frequency of 0.7118 THz, which is close to the LPR of a single resonator. This phenomenon demonstrates a strong coherent coupling between the LPR of the VO_2_–silicon resonator and second-order SLR modes; it is also the key to the generation of ultra-high *Q*-factor.^36^Fig. 2(b) demonstrates the variation in the Fano resonance at different periods (*P*). It can be observed that the Fano resonance shifts to lower frequencies as the period increases, which further proves that the Fano resonance is affected by the SLR mode.

**Fig. 3:**
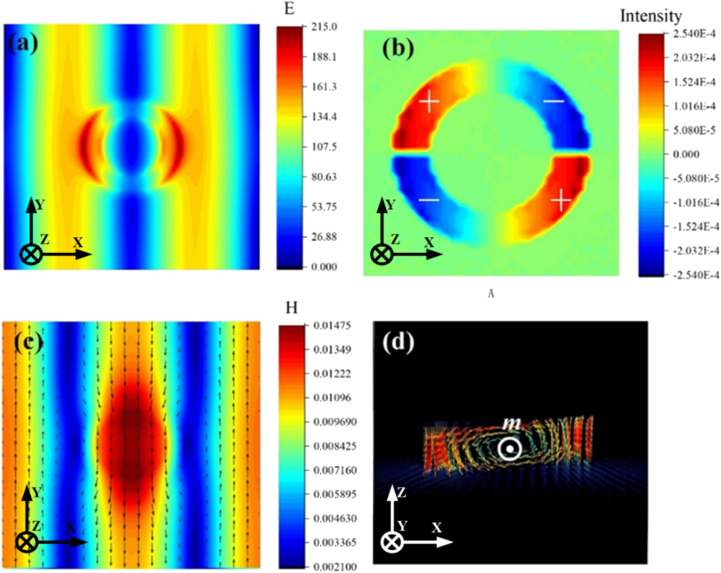
(a) Electric field distribution, (b) charge distribution, (c) magnetic field distribution and (d) *x*–*z* cross-sectional current in the proposed design.

To gain a deeper understanding of the physical mechanisms of Fano resonance, we simulate and analyze the distribution of electric field, magnetic field, current and charge at Fano resonance frequency. In Fig. 3(a), we see that there is an obvious enhancement of the electric field on both sides of the ring resonator. In addition, we can see that along with the excitation of the second-order (1,1) lattice resonance in the absence of a resonator unit, the two sides of the substrate still produce an obvious electric field resonance, indicating that the electric quadrupole is excited along with the second-order (1,1) SLR. In Fig. 3(b), we can see that the charge distribution on the ring is “+ − + −” along four clockwise parts, which can be recognized as an electric quadrupole resonance mode. In Fig. 3(c), we can see that a significant magnetic field enhancement occurs in each of the resonator units, exciting a magnetic resonance that was constrained by a strong field on the surface of the substrate. Furthermore, according to the propagation direction of the magnetic field at the Fano resonance frequency, the magnetic field of a single resonator propagates in the opposite direction to that in the substrate, which in turn generates interfering couplings at the second-order SLR. Therefore, the electromagnetic wave energy can be efficiently confined to the metamaterial without combining with the free space. Fig. 3(d) illustrates the current distribution in the *x*–*z* cross-section of the metamaterial; it can be seen that currents with opposite vector directions are formed on both sides of the resonator and circulate inside the resonator. The presence of such circulating currents causes the resonator to produce a magnetic dipole moment *m* on the *y*-axis, as shown in Fig. 3(c).^37,38^ The multipole expansion method shows that for magnetic dipole resonances, the radiation losses are influenced by the magnetic moment *m* or equivalent current element *J*. Since currents on both sides of the ring resonator are in opposite directions, forming a cancellation, the equivalent current is almost zero. Thus, almost no radiation is lost, which in turn contributes to the formation of the ultra-high *Q*-factor Fano resonance.^6^

From the above analysis, it is concluded that the scattered field excited at each metamaterial unit resonates at the substrate surface and produces interference phenomena, leading to in-plane confinement of the scattered field and reduction of radiation losses. The coupling of the magnetic dipole and electric quadrupole to the SLR confines the electromagnetic energy in the lattice, which greatly suppresses radiative damping and ultimately leads to a high *Q*-factor of more than 10^6^ in the terahertz band.

To quantitatively analyze the ultra-high *Q*-factor Fano resonance mechanism, we calculated the radiative components of the magnetic dipole (*m*), magnetic quadrupole (*Q*_m_), electric quadrupole (*Q*_e_), electric dipole (*P*) and toroidal dipole (*T*) using eqn (3) (ref. 39 and 40)3
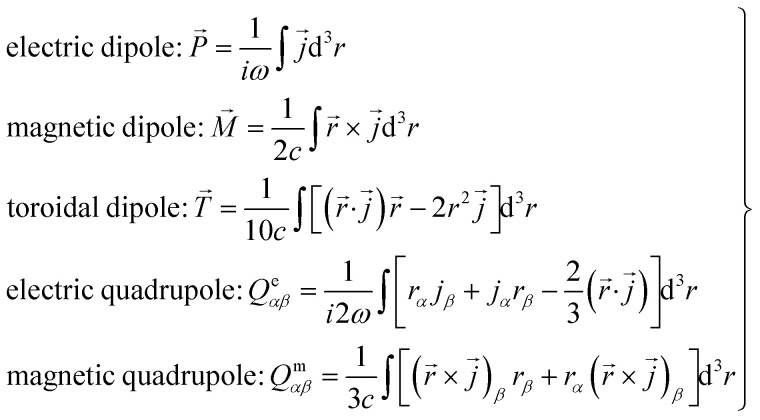


**Fig. 4:**
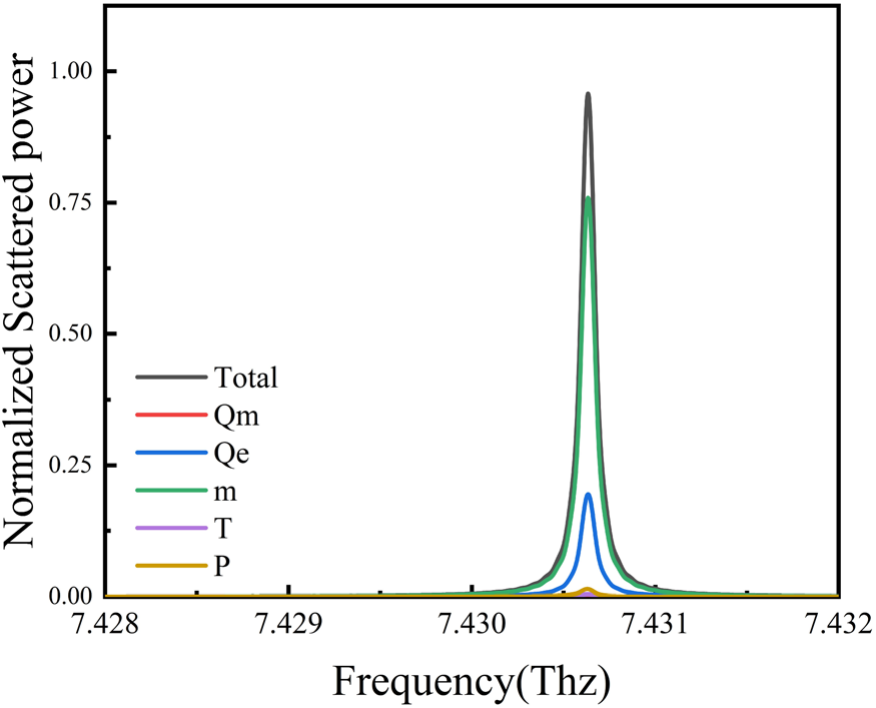
Scattering powers by multipolar decomposition of the proposed metamaterial.

Fig. 4 shows the results of the multipole analysis, where modes excited at the Fano resonance frequency are mainly magnetic dipole resonance and electric quadrupole resonance; these observations are consistent with those shown in Fig. 3(c) and (d), and thus, magnetic resonance is dominant at the Fano resonance frequency.

**Fig. 5:**
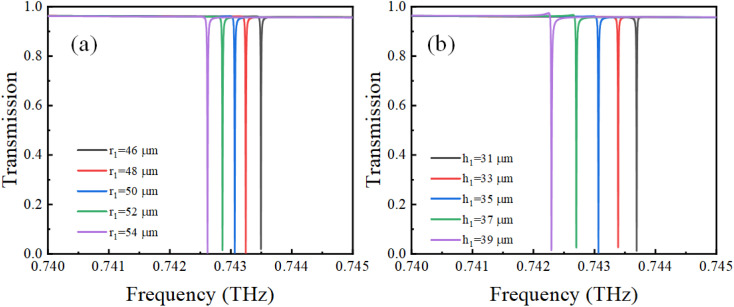
Transmission spectra for different (a) inner ring radii (*r*_1_) and (b) ring heights (*h*_1_).

The parameters of the structure have an important influence on the performance of magnetic resonance. The effect of fabrication defects on the magnetic resonance must be considered in the actual preparation process. Fig. 5(a) demonstrates the effect of transmission spectra for different inner ring radii (*r*_1_) while keeping a constant ring width. It was found that the Fano resonance frequency gradually moves to lower frequencies with the increase in the inner ring radius. In Fig. 5(b), it is shown that as the silicon ring height increases, the Fano resonance frequency also exhibits a shift toward lower frequencies. This is because in the plasma resonance mode, the resonance frequency can be effectively controlled by the properties and structural parameters of the material.^41^

**Fig. 6:**
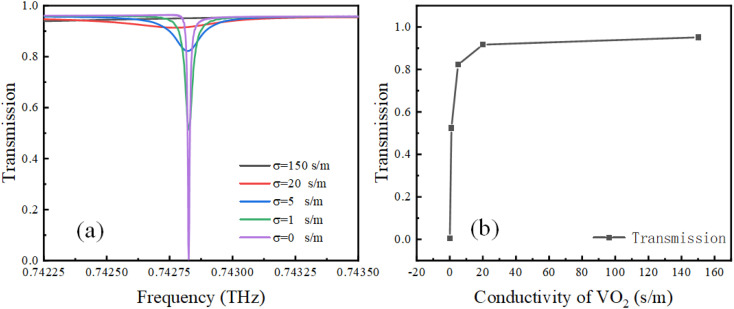
(a) Fano resonance transmission with different conductivities of VO_2_, (b) relationship between conductivity and transmittance at the lowest point of Fano resonance.

As a phase change material, vanadium dioxide can undergo phase change from an insulator state to a metallic state by various methods, such as temperature, light, and electric field, thereby producing an adjustable conductivity of VO_2_.^42,43^ As shown in Fig. 6(a), the amplitude of the Fano resonance decreases as the VO_2_ conductivity (*σ*) decreases, and the Fano resonance almost disappears, when the conductivity is set at 150 s m^−1^. To demonstrate the impact of VO_2_ conductivity on Fano resonance more clearly, Fig. 6(b) shows the relationship between conductivity and transmittance at the lowest point of Fano resonance. Therefore, this design achieves an optical switching function, where the modulation depth can be up to 98.9% using MD = [(*T*_max_ − *T*_min_)/(*T*_max_ + *T*_min_)] × 100%.

**Fig. 7:**
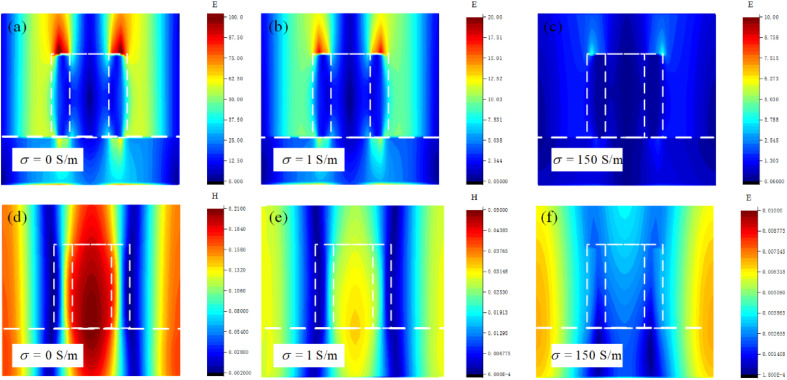
Distribution of (a)–(c) electric and (d)–(f) magnetic fields in the *x*–*z* cross-section of metamaterial with different conductivities of VO_2_.

To clarify the physical mechanism of this amplitude tunability, in Fig. 7, we show electric and magnetic fields calculated for the *x*–*z* cross-section of the metamaterial cell. From electric field distributions in Fig. 7(a–c), it can be observed that the electric field enhancement at the ring resonator gradually diminishes as the conductivity increases, and it disappears almost completely when the conductivity is 150 S m^−1^. According to magnetic field distributions in Fig. 7(d and e), when the VO_2_ conductivity is 0 S m^−1^, magnetic resonance is excited at the center of the ring resonator. As the conductivity increases, the magnetic resonance at the center of the ring weakens significantly. When the conductivity is 150 S m^−1^, the magnetic resonance almost disappears. This phenomenon illustrates that as the conductivity of VO_2_ increases, the excitation of the electrical and magnetic resonance sat the ring resonator ring are affected and weaken the coupling between the LPR and SLR, which ultimately leads to the disappearance of the ultra-high *Q*-factor Fano resonance. Here, it can be concluded that the increase in VO_2_ conductivity of the proposed metamaterial weakens the intensity of Fano resonance, resulting in the gradual disappearance of the Fano line in Fig. 6(a).

Enhancement of the electrical and magnetic fields on the surface of a metamaterial sensor can increase the interaction of incident electromagnetic waves with the analyte, which in turn improves the sensitivity of the sensor to the medium refractive index (RI). The ring-shaped resonator array proposed in our design can be considered a frequency-selective metasurface. Therefore, the LPR resonance frequency is estimated using eqn (4).^44^4
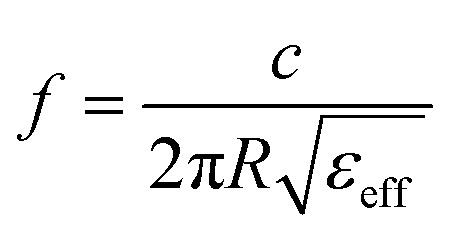
where *R* is the equivalent radius of the ring resonator; *ε*_eff_ can be expressed as the equivalent refractive index of the medium around the resonator, and *c* denotes the velocity of the incident wave. It can be inferred that with an increase in the refractive index of the analyte, which leads to an increase in the overall equivalent refractive index of the medium around the resonator, the LPR resonance frequency *f* decreases. Eventually, the Fano resonance frequency achieved by the coupling of LPR and SLR also decreases.

**Fig. 8:**
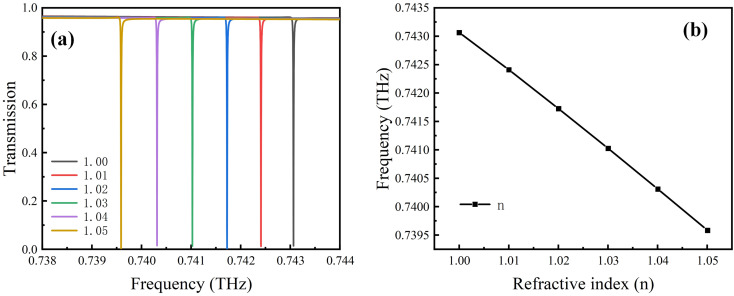
(a) Transmission and (b) Fano resonance frequency at different refractive indices of the analyte.

Due to the extremely narrow Fano resonance peak that can be achieved by our design, the resonance peak frequency will significantly change when the analyte RI changes. As shown in Fig. 8(a), as the analyte RI increases, the Fano resonance gradually moves to lower frequencies.

The influence of the analyte RI on the Fano resonance frequency is shown in Fig. 8(b); it can be seen that there is a good linear relationship between frequency change and RI, which is favorable for the application of this metamaterial as a sensor. The sensitivity is calculated to be 0.06952 Thz per RIU using the relation, *S* = Δ*f*/Δ*n*. The FoM can be calculated using the equation, FOM = *S*/FWHM = *S* × *Q*/*f*, where FWHM is the full width at half maximum, and *f* refers to the resonance frequency. The present design can achieve a FoM value of 15 456.^45^

In Table 1, we summarize relevant sensors using metamaterials.^41,46–48^ It can be concluded that our proposed design has excellent performance (ultra-high *Q*-factor and FOM). In addition, our design can realize the optical switching function with modulation depths up to 98.8% by tuning the VO_2_ conductivity. The designed structure is also relatively simple and easy to prepare for future experiments.

**Table 1:** Comparison of our work with previously proposed terahertz metamaterial sensors

Material	FOM	Sensitivity	*Q* factor	Reference
Metal	—	734 GHz per RIU	>10^4^	41
GaAs	392	1762 GHz per RIU	444	46
Metal	63.83	379 GHz per RIU	66.01	47
Graphene and LiTaO_3_	121.39	309.54 GHz per RIU	—	48
VO_2_ and silicon	15 456	69.52 GHz per RIU	165 198	This work

## 4. Conclusions

In summary, we theoretically designed a metamaterial consisting of a periodic arrangement of VO_2_–silicon ring resonators, which is capable of achieving sensing functionality with an ultra-high *Q*-factor, along with optical switching functions with high modulation depth. It is demonstrated by electric field, magnetic field, current analysis and multipole analysis that the proposed metamaterial can excite obvious magnetic dipole resonance and electric quadrupole resonance. Coupling of the above two resonant modes to the SLR suppresses radiation loss and achieves a *Q*-factor as high as 165 198. The Fano resonance frequency can be adjusted by changing the parameters of the resonance units of the proposed metamaterial. Due to the tunable conductivity of VO_2_, the metamaterial is also capable of optical switching with a modulation depth of 98.8%. In addition, we calculated the theoretical sensitivity to show that our design can realize a sensitivity of 69.52 Ghz per RIU and a high FoM of 15 456. The proposed scheme provides a path to achieve tunable terahertz functional devices with an ultra-high *Q*-factor. The findings of this investigation are of great significance for the study of the suppression of radiation loss and enhancement of terahertz field interactions with matter.

## Conflicts of interest

There are no conflicts to declare.

## Supplementary Material
